# COVID-19 Vaccine–Related Attitudes and Beliefs in Canada: National Cross-sectional Survey and Cluster Analysis

**DOI:** 10.2196/30424

**Published:** 2021-12-23

**Authors:** Jamie L Benham, Omid Atabati, Robert J Oxoby, Mehdi Mourali, Blake Shaffer, Hasan Sheikh, Jean-Christophe Boucher, Cora Constantinescu, Jeanna Parsons Leigh, Noah M Ivers, Scott C Ratzan, Madison M Fullerton, Theresa Tang, Braden J Manns, Deborah A Marshall, Jia Hu, Raynell Lang

**Affiliations:** 1 Department of Medicine University of Calgary Calgary, AB Canada; 2 Department of Community Health Sciences University of Calgary Calgary, AB Canada; 3 Department of Economics University of Calgary Calgary, AB Canada; 4 Haskayne School of Business University of Calgary Calgary, AB Canada; 5 Department of Family and Community Medicine University of Toronto Toronto, ON Canada; 6 School of Public Policy and Department of Political Sciences University of Calgary Calgary, AB Canada; 7 School of Health Administration Dalhousie University Halifax, NS Canada; 8 Institute for Health System Solutions and Virtual Care Women's College Hospital Toronto, ON Canada; 9 School of Public Health and Health Policy City University of New York New York, NY United States

**Keywords:** coronavirus, COVID-19, public health, marketing, behavior, risk reduction, attitudes, compliance, vaccine, hesitancy, risk, belief, communication, cross-sectional, Canada, gender, education, income, race, ethnicity

## Abstract

**Background:**

There are concerns that vaccine hesitancy may impede COVID-19 vaccine rollout and prevent the achievement of herd immunity. Vaccine hesitancy is a delay in acceptance or refusal of vaccines despite their availability.

**Objective:**

We aimed to identify which people are more and less likely to take a COVID-19 vaccine and factors associated with vaccine hesitancy to inform public health messaging.

**Methods:**

A Canadian cross-sectional survey was conducted in Canada in October and November 2020, prior to the regulatory approval of the COVID-19 vaccines. Vaccine hesitancy was measured by respondents answering the question “what would you do if a COVID-19 vaccine were available to you?” Negative binomial regression was used to identify the factors associated with vaccine hesitancy. Cluster analysis was performed to identify distinct clusters based on intention to take a COVID-19 vaccine, beliefs about COVID-19 and COVID-19 vaccines, and adherence to nonpharmaceutical interventions.

**Results:**

Of 4498 participants, 2876 (63.9%) reported COVID-19 vaccine hesitancy. Vaccine hesitancy was significantly associated with (1) younger age (18-39 years), (2) lower education, and (3) non-Liberal political leaning. Participants that reported vaccine hesitancy were less likely to believe that a COVID-19 vaccine would end the pandemic or that the benefits of a COVID-19 vaccine outweighed the risks. Individuals with vaccine hesitancy had higher prevalence of being concerned about vaccine side effects, lower prevalence of being influenced by peers or health care professionals, and lower prevalence of trust in government institutions.

**Conclusions:**

These findings can be used to inform targeted public health messaging to combat vaccine hesitancy as COVID-19 vaccine administration continues. Messaging related to preventing COVID among friends and family, highlighting the benefits, emphasizing safety and efficacy of COVID-19 vaccination, and ensuring that health care workers are knowledgeable and supported in their vaccination counselling may be effective for vaccine-hesitant populations.

## Introduction

In the fall of 2020, regions of Canada were experiencing a second wave of COVID-19 with rising case counts, hospitalizations, and deaths [1]. Although several vaccines against SARS-CoV-2 were in development [2], they were not yet available as public health tools to mitigate the spread of COVID-19, as the first COVID-19 vaccine was not authorized by Health Canada until December 9, 2020 [2]. This meant that in the fall of 2020, nonpharmacologic interventions (NPIs), including practicing physical distancing, wearing a face mask if physical distancing is not possible, staying home when sick, and limiting large gatherings were the only means to reduce the transmission of COVID-19 [3].

Although there was great optimism about the potential emergence of safe and effective vaccines against SARS-CoV-2, COVID-19 vaccine hesitancy was becoming evident in the summer and fall of 2020 [4-7]. Vaccine hesitancy is defined by the Strategic Advisory Group of Experts (SAGE) Working Group as a “delay in acceptance or refusal of vaccination despite availability of vaccination services” [8]. The reasons for vaccine hesitancy are heterogeneous and complex [9]. The SAGE Working Group created a framework of vaccine hesitancy determinants, which consists of 3 domains: (1) contextual influences (eg, socioeconomic group, political climate), (2) individual and social group influences (eg, social norm, personal experience), and (3) vaccine characteristics (eg, perceived risks and benefits, health care provider attitudes) [8]. This framework can be used to determine the potential factors contributing to vaccine hesitancy with respect to a COVID-19 vaccine within the unique context of the COVID-19 pandemic.

Several studies have looked at the risk factors for vaccine hesitancy in populations around the world and have found that many different factors, including sociodemographic variables and concerns about efficacy and safety of COVID-19 vaccines, may contribute to COVID-19 vaccine hesitancy [10]. It has been noted, however, that factors associated with vaccine hesitancy identified in the general population may not be consistent with factors associated with vaccine hesitancy in specific subpopulations [11]. Therefore, to improve overall vaccine uptake, it is important to examine the risk factors for vaccine hesitancy in the specific population segments who report increased vaccine hesitancy.

In the summer of 2020, we designed a mixed methods study to examine COVID-19 attitudes, beliefs, and behaviors among Canadians with an overreaching goal of informing targeted public health messaging to improve adherence to NPIs and vaccine uptake. We have previously published the initial phases of this mixed methods study including a pilot survey [5] and a qualitative study [4]. This preliminary work found that there were mixed views regarding willingness to take a COVID-19 vaccine and identified a number of risk factors with respect to COVID-19 vaccine hesitancy, including low perceived risk of COVID-19 infection, vaccine-specific concerns, low adherence to NPIs, and sources of COVID-19 information [4,5].

Based on the findings from the initial work in our mixed methods study [4,5], we designed a national survey to further explore the risk factors for vaccine hesitancy to identify segmented populations of individuals with vaccine hesitancy to inform targeted public health messaging campaigns. The objectives of this study were to (1) identify which groups of people are more or less likely to take a COVID-19 vaccine among Canadian adults, (2) determine which attitudes toward COVID-19 are associated with vaccine hesitancy, (3) determine if vaccine hesitancy is associated with adherence to NPIs for COVID-19, and (4) evaluate the relationship between persons’ vaccine attitudes and their sources of COVID-19 information.

## Methods

### Study Design, Participants, and Setting

We used a cross-sectional survey to assess the attitudes and beliefs about vaccines and vaccine hesitancy among adults aged 18 years or older living in Canada. The survey was administered online by the Angus Reid Institute [12], a national, not-for-profit, research foundation, from October 27 to November 2, 2020, as the preliminary data on vaccine efficacy and safety of the COVID-19 vaccine, Pfizer-BioNTech, was being submitted to Health Canada for review but before it was approved for use in Canada on December 9, 2020. Survey participants were drawn randomly from the Angus Reid Forum and contacted electronically. The Angus Reid Forum is comprised of 70,000 individuals from across Canada designed to be representative of the Canadian population with sociodemographic characteristics verified to match electoral and census data in each sampling region [12]. To obtain a sample size of 4500, the survey was distributed to 14,887 potential participants. Sampling was stratified for equal representation of Alberta residents and residents of the other Canadian provinces combined. This sampling strategy was used to allow for comparison of 2 Canadian applications used to facilitate contact tracing, that is, ABTraceTogether (a contact tracing application, which is only available in Alberta) and COVID Alert (an exposure notification application available in 8 provinces and the Northwest territories). A copy of the survey questions that were administered can be found in Multimedia Appendix 1.

This study was approved by the Conjoint Health Research Ethics Board at the University of Calgary (REB20-1228). Informed consent was obtained from each participant prior to commencing the survey, and participation was voluntary. Responses were deidentified at the time of collection to ensure participant anonymity and privacy. If participants started the survey but did not complete it, it was assumed that consent was withdrawn and their survey responses were not saved. Consistent with Angus Reid Forum policy [12], members of the Angus Reid Forum who completed the survey received a small monetary incentive. The Strengthening Reporting of Observational Studies in Epidemiology (STROBE) checklist was used to report our findings [13].

### Outcome Measure

The main outcome measure was vaccine hesitancy. Survey participants were asked what they would do if a COVID-19 vaccine were available to them and given the following 4 options: (1) get a vaccine as soon as possible, (2) eventually get a vaccine, but wait a while first, (3) not get a vaccine, or (4) not sure. Vaccine hesitancy was defined as any of the latter 3 responses consistent with the SAGE Working Group definition of vaccine hesitancy [8].

### Risk Factors for Vaccine Hesitancy

We considered factors that could be associated with vaccine hesitancy in each of the domains of the SAGE framework (contextual influences, individual and group influences, and vaccine-specific factors) [8] based on a review of the literature, focus groups [4], and a pilot survey [5] that we completed in Alberta, Canada in the summer of 2020. For contextual influences, we determined demographic factors, including sex, age, geographical region, household income, highest level of education, ethnicity, and political leaning. In terms of individual and group influences, we determined participants’ attitudes toward COVID-19 and the COVID-19 vaccine, adherence to NPIs (ie, physical distancing, masking, reducing interactions with others, staying home when sick), trusted sources of COVID-19 information, and trusted institutions. For vaccine characteristics, participants were asked about the perceived risks and benefits of COVID-19 vaccines.

### Statistical Analysis

Descriptive statistics (percentage frequencies) were calculated for all participant characteristics, adherence to NPIs, attitudes toward COVID-19 and COVID-19 vaccines, and trusted sources of COVID-19 information. Respondents were excluded if they did not answer all survey questions, and therefore, there were no missing data. Negative binomial regression models were used to estimate crude prevalence ratios (PRs) for factors associated with being vaccine hesitant compared to not being vaccine hesitant. Each PR was reported with the associated 95% CI. We used multiple models to examine the association between vaccine hesitancy and each of the following: (1) sociodemographic characteristics, (2) attitudes toward COVID-19 vaccine, (3) adherence toward NPIs, (4) attitudes toward COVID-19, and (5) trusted sources of COVID-19 information. We also calculated adjusted prevalence ratios (aPRs) by using sociodemographic characteristics identified through a literature search [10,14-16] as being associated with vaccine hesitancy, including sex at birth, age, ethnicity, province of residence, education, household income, and political leaning.

To identify data-driven patterns in survey responses with respect to vaccine hesitancy, we used cluster analysis. The cluster analysis was based on intention to take a COVID-19 vaccine, beliefs about COVID-19 and COVID-19 vaccine, and adherence to NPIs. The K-means algorithm was used to partition the data set into distinct clusters. This iterative algorithm assigns observations to a cluster such that within each cluster, the sum of the squared distance between observations and the arithmetic mean of all observations is minimized. Cluster analysis was used to integrate COVID-19 vaccine intention, COVID-19 beliefs, and adherence to NPIs into similar like-minded groupings to identify insights that can be utilized for targeted messaging and interventions. By using several exposures to establish these clusters, we aimed to create clusters with greater similarity in motivations and attitudes for vaccine intention and gain a deeper understanding of vaccine hesitancy. Negative binomial regression was used to estimate crude PRs and 95% CI comparing sociodemographic characteristics between each of the clusters with cluster 2 as the reference. Analyses were conducted using STATA Version 15.1 (Stata Corp). A *P* value of <.05 was set as significant.

## Results

### Survey Participation

Of the 14,887 survey invitations distributed, 5893 (39.6%) invitations were accepted in the 7 days the survey was available. Of those, 4498 (76.3%) participants completed the survey and were included in the analysis (Table 1), while 1395 (23.7%) participants were excluded owing to one or more incomplete responses. Participants who completed the survey were similar to those who started but did not complete the survey in terms of sex, age, province of residence, highest level of education, and ethnicity.

**Table 1:** Participant characteristics and association with COVID-19 vaccine hesitancy in October to November 2020 (N=4498).

Characteristic			Total, n (%)		Vaccine hesitancy, n (%)					Prevalence ratio^a^ (95% CI)			Adjusted prevalence ratio^b^ (95% CI)
					No		Yes						
Participants			4498 (100)		1622 (36.1)		2876 (63.9)		N/A^c^			N/A	
**Sex at birth**													
	Female	2294 (51)		815 (35.5)		1479 (64.5)		Ref^d^			Ref		
	Male	2204 (49)		807 (36.6)		1397 (63.4)		0.98 (0.91-1.06)			0.93 (0.86-1.01)		
**Age (years)**													
	18-34	1341 (29.8)		505 (37.7)		836 (62.3)		Ref			Ref		
	35-54	1585 (35.2)		504 (31.8)		1081 (68.2)		1.09 (1.00-1.20)			1.04 (0.95-1.14)		
	55+	1572 (35)		613 (39)		959 (61)		0.98 (0.89-1.07)			0.90 (0.82-0.99)		
**Province of residence**													
	Alberta	1998 (44.4)		672 (33.6)		1326 (65.4)		Ref			Ref		
	British Columbia	502 (11.2)		176 (35.1)		326 (64.9)		0.98 (0.87-1.10)			1.04 (0.92-1.17)		
	Prairie provinces^e^	445 (9.9)		156 (35.1)		259 (58.2)		0.98 (0.76-1.11)			0.95 (0.84-1.08)		
	Ontario	800 (17.8)		311 (38.9)		489 (61.1)		0.92 (0.83-1.02)			0.96 (0.87-1.07)		
	Quebec	502 (11.2)		203 (40.4)		299 (59.6)		0.90 (0.79-1.02)			0.97 (0.85-1.10)		
	Atlantic provinces^e^	251 (5.6)		104 (41.4)		147 (58.6)		0.88 (0.74-1.05)			0.95 (0.80-1.13)		
**Household income^f^ (CAD)**													
	<$50,000	1030 (22.9)		342 (33.2)		688 (66.8)		Ref			Ref		
	$50,000-$99,999	1353 (30.1)		486 (35.9)		867 (64.1)		0.96 (0.87-1.06)			0.97 (0.88-1.08)		
	$100,000-$199,999	1300 (28.9)		511 (39.3)		789 (60.7)		0.91 (0.82-1.01)			0.93 (0.84-1.04)		
	≥$200,000	229 (5.1)		102 (44.5)		127 (55.5)		0.83 (0.69-1.00)			0.85 (0.70-1.03)		
	Rather not say	586 (13)		181 (30.9)		405 (69.1)		1.03 (0.92-1.17)			1.02 (0.90-1.15)		
**Highest level of education**													
	High school graduate or less	897 (19.9)		256 (28.5)		641 (71.5)		Ref			Ref		
	Some college or trade school	840 (18.7)		240 (28.6)		600 (71.4)		1.00 (0.89-1.12)			1.01 (0.90-1.13)		
	College or trade school	996 (22.1)		301 (30.2)		695 (69.8)		0.98 (0.88-1.09)			0.98 (0.88-1.10)		
	Some university	454 (10.1)		185 (40.7)		269 (59.3)		0.83 (0.72-0.96)			0.85 (0.73-0.97)		
	University degree	1311 (29.1)		640 (48.8)		671 (51.2)		0.72 (0.64-0.80)			0.73 (0.65-0.81)		
**Ethnicity**													
	Caucasian	3862 (85.9)		1430 (37)		2432 (63)		Ref			Ref		
	Indigenous/First Nations/Metis/Inuit	228 (5)		68 (29.8)		160 (70.2)		1.11 (0.95-1.31)			1.09 (0.93-1.27)		
	Asian	193 (4.3)		65 (33.7)		128 (66.3)		1.05 (0.88-1.26)			1.15 (0.96-1.37)		
	Caribbean/African/South American	70 (1.6)		19 (27.1)		51 (72.9)		1.16 (0.88-1.53)			1.16 (0.88-1.54)		
	Other	145 (3.2)		40 (27.6)		105 (72.4)		1.15 (0.95-1.40)			1.10 (0.91-1.34)		
**Political leaning**													
	Liberal	1841 (40.9)		936 (50.8)		905 (49.2)		0.73 (0.66-0.80)			0.74 (0.67-0.82)		
	Moderate/middle of the road	1029 (22.9)		334 (32.5)		695 (67.5)		Ref			Ref		
	Conservative	1628 (36.2)		352 (21.6)		1276 (78.4)		1.16 (1.06-1.27)			1.18 (1.07-1.29)		

^a^Prevalence ratio is the prevalence of vaccine hesitancy compared with the prevalence of planning to take a COVID-19 vaccine.

^b^Adjusted for sex, age, province of residence, household income, education level, ethnicity, and political leaning.

^c^N/A: not applicable.

^d^Ref: reference value.

^e^Prairie provinces include Saskatchewan and Manitoba; Atlantic provinces include Nova Scotia, New Brunswick, Prince Edward Island, and Newfoundland and Labrador.

^f^CAD $1=US $0.75.

### Participant Characteristics

Participant demographic and socioeconomic characteristics are presented in Table 1. The majority of the participants were females (2294/4498, 51%) and Caucasian (3862/4498, 85.9%). The mean participant age was 47 (SD 16) years with participant ages ranging from 18 to 94 years. The majority of the participants indicated that they were vaccine hesitant and reported they would delay taking a COVID-19 vaccine when offered one (1817/4498, 40.4%), not take a COVID-19 vaccine (708/4498, 15.7%), or were not sure about taking a COVID-19 vaccine (351/4498, 7.8%). The remaining one-third (1622/4498, 36.1%) of the participants reported that they would take a COVID-19 vaccine as soon as possible. Participants aged 55 years or older had lower prevalence of vaccine hesitancy compared with those aged 18-34 years (aPR 0.90, 95% CI 0.82-0.99). University education was also associated with lower prevalence of vaccine hesitancy. Compared with participants who reported their highest level of education as high school graduate or less, the adjusted prevalence was 0.85 (95% CI 0.73-0.97) for some university education and 0.73 (95% CI 0.65-0.81) for participants who had completed a university degree. Liberal political leaning was associated with lower prevalence of vaccine hesitancy compared with participants who reported moderate or middle of the road political leaning (aPR 0.74, 95% CI 0.67-0.82), while conservative political leaning was associated with higher prevalence of vaccine hesitancy (aPR 1.18, 95% CI 1.07-1.29). Biological sex, household income, ethnicity, and province of residence were not associated with vaccine hesitancy.

### Attitudes Toward COVID-19 Vaccine

More than half of the participants (2501/4498, 55.6%) felt that the benefits of taking a vaccine outweigh its risks, while 969 (22%) were unsure and 1028 (22%) disagreed (Table 2).

**Table 2:** Associations between COVID-19 vaccine hesitancy and attitudes toward COVID-19 vaccines in October to November 2020 (N=4498).

				Total, n (%)		Vaccine hesitancy, n (%)				Prevalence ratio^a^ (95% CI)			Adjusted prevalence ratio^b^ (95% CI)
						No		Yes					
Participants				4498 (100)		1622 (36.1)		2876 (63.9)		N/A^c^		N/A	
**Attitudes toward COVID-19 vaccines**													
	**Would take a vaccine to protect family**												
		Agree	3293 (73.2)		1603 (48.7)		1690 (51.3)		Ref^d^		Ref		
		Disagree	810 (18)		17 (2.1)		793 (97.9)		1.91 (1.75-2.08)		1.77 (1.62-1.94)		
		Not sure	395 (8.8)		2 (0.5)		393 (99.5)		1.94 (1.74-2.16)		1.85 (1.66-2.07)		
	**A vaccine will end the pandemic**												
		Agree	1265 (28.1)		691 (54.6)		574 (45.4)		Ref		Ref		
		Disagree	2214 (49.2)		587 (26.5)		1627 (73.5)		1.62 (1.47-1.78)		1.54 (1.40-1.70)		
		Not sure	1019 (22.7)		344 (33.8)		675 (66.2)		1.46 (1.31-1.63)		1.43 (1.28-1.61)		
	**Usually get the flu vaccine**												
		Agree	2523 (56.1)		1301 (51.6)		1222 (48.4)		Ref		Ref		
		Disagree	1901 (42.3)		301 (15.8)		1600 (84.2)		1.74 (1.61-1.87)		1.67 (1.55-1.80)		
		Not Sure	74 (1.6)		20 (27)		54 (73)		1.51 (1.15-1.98)		1.50 (1.14-1.97)		
	**Concern about short-term side effects**												
		Agree	2583 (57.4)		533 (20.6)		2050 (79.4)		Ref		Ref		
		Disagree	1443 (32.1)		922 (63.9)		521 (36.1)		0.45 (0.41-0.50)		0.47 (0.43-0.52)		
		Not Sure	472 (10.5)		167 (35.4)		305 (64.6)		0.81 (0.72-0.92)		0.83 (0.73-0.93)		
	**Concern about long-term side effects**												
		Agree	2703 (60.1)		542 (20.1)		2161 (79.9)		Ref		Ref		
		Disagree	1294 (28.8)		881 (68.1)		413 (31.9)		0.40 (0.36-0.44)		0.42 (0.38-0.46)		
		Not sure	501 (11.1)		199 (39.7)		302 (60.3)		0.75 (0.67-0.85)		0.78 (0.69-0.88)		
	**Vaccine developed too fast**												
		Agree	1985 (44.1)		162 (8.2)		1823 (91.8)		Ref		Ref		
		Disagree	1874 (41.7)		1248 (66.6)		626 (33.4)		0.36 (0.33-0.40)		0.38 (0.35-0.42)		
		Not sure	639 (14.2)		212 (33.2)		427 (66.8)		0.73 (0.65-0.81)		0.75 (0.67-0.83)		
	**Vaccine benefits outweigh the risks**												
		Agree	2501 (55.6)		1457 (58.3)		1044 (41.7)		Ref		Ref		
		Disagree	1028 (22.9)		40 (3.9)		988 (96.1)		2.30 (2.11-2.51)		2.17 (1.98-2.38)		
		Not sure	969 (21.5)		125 (12.9)		844 (87.1)		2.09 (1.91-2.28)		2.02 (1.85-2.22)		
	**Would take vaccine if family/friends do**												
		Agree	1681 (37.4)		745 (44.3)		936 (55.7)		Ref		Ref		
		Disagree	2296 (51)		717 (31.2)		1579 (68.8)		1.24 (1.14-1.34)		1.17 (1.08-1.27)		
		Not sure	521 (11.6)		160 (30.7)		361 (69.3)		1.24 (1.10-1.41)		1.21 (1.07-1.37)		
	**Would take vaccine if advised by family doctor/pharmacist/public health official**												
		Agree	2775 (61.7)		1422 (51.2)		1353 (48.8)		Ref		Ref		
		Disagree	1319 (29.3)		131 (10)		1188 (90.1)		1.85 (1.71-2.00)		1.73 (1.59-1.87)		
		Not sure	404 (9)		69 (17.1)		335 (82.9)		1.70 (1.51-1.92)		1.65 (1.46-1.86)		

^a^Prevalence ratio is the prevalence of vaccine hesitancy compared with the prevalence of planning to take a COVID-19 vaccine.

^b^Adjusted for sex, age, province of residence, household income, education level, ethnicity, and political leaning.

^c^N/A: not applicable.

^d^Ref: reference value.

Those who disagreed had higher prevalence of vaccine hesitancy compared with those who agreed (aPR 2.17, 95% CI 1.98-2.38; Table 2). Opinions were mixed on whether a COVID-19 vaccine would end the pandemic with 1265 (28.1%) in agreement, 2214 (49.2%) in disagreement, and 1019 (22.7%) being undecided. Participants who disagreed that the vaccine would end the pandemic had a higher prevalence of vaccine hesitancy than those who agreed (aPR 1.54, 95% CI 1.40-1.70).

Participants reported that they would be more likely to take a COVID-19 vaccine if it was recommended by a family doctor, pharmacist, or public health nurse (2775/4498, 61.7%) or if their friends or family took a vaccine (1681/4498, 37.4%). However, the prevalence of vaccine hesitancy was higher in participants who disagreed that they would take a vaccine if their friends/family do (aPR 1.17, 95% CI 1.08-1.27) or if it was recommended by a family doctor, pharmacist, or public health nurse (aPR 1.73, 95% CI 1.59-1.87). Numerous participants (3293/4498, 73.2%) said they would take a COVID-19 vaccine to protect their family; participants who disagreed with this statement had a higher prevalence of vaccine hesitancy compared with those who agreed (aPR 1.77, 95% CI 1.62-1.94). Many participants were concerned about the short-term side effects (2583/4498, 57.4%) and long-term side effects (2703/4498, 60.1%). Participants (1874/4498, 41.7%) who disagreed with the statement that vaccines were developed too fast had a lower prevalence of vaccine hesitancy compared with those who agreed (aPR 0.38, 95% CI 0.35-0.42).

### NPIs

The majority of the participants reported physical distancing (3782/4498, 84.1%), wearing face masks (3873/4498, 86.1%), avoiding crowded spaces (3517/4498, 78.2%), and staying home when sick (3857/4498, 85.7%) all or most of the time (Table 3). Participants who reported only adhering to any of these NPIs sometimes, rarely, or never had higher odds of vaccine hesitancy. Participants who reported rarely or never wearing a face mask had an adjusted prevalence of vaccine hesitancy of 1.38 (95% CI 1.22-1.56) compared with those who reported wearing a face mask all the time or most of the time. For physical distancing, those who reported adhering to this NPI sometimes had higher prevalence of vaccine hesitancy than those who practiced physical distancing all the time or most of the time (aPR 1.32, 95% CI 1.18-1.48). Compared with those who reported avoiding crowded spaces all the time or most of the time, participants who reported rarely or never avoiding public spaces had higher prevalence of vaccine hesitancy (aPR 1.35, 95% CI 1.21-1.50).

**Table 3:** Associations between COVID-19 vaccine hesitancy, adherence to public health measures, and attitudes toward COVID-19 in October to November 2020 (N=4498).

Characteristic			Total, n (%)	Vaccine hesitancy, n (%)		Prevalence ratio^a^ (95% CI)	Adjusted prevalence ratio^b^ (95% CI)
				No	Yes		
Participants			4498 (100)	1622 (36.1)	2876 (63.9)	N/A^c^	N/A
**Adherence to nonpharmaceutical interventions**							
	**Physical distancing**						
		All the time/most of the time	3777 (84)	1523 (40.3)	2254 (69.7)	Ref^d^	Ref
		Sometimes	457 (10.2)	68 (14.9)	389 (85.1)	1.43 (1.28-1.59)	1.32 (1.18-1.48)
		Rarely/never	264 (5.8)	31 (11.7)	233 (88.3)	1.48 (1.29-1.69)	1.31 (1.13-1.50)
	**Wearing face masks**						
		All the time/most of the time	3868 (86)	1566 (40.5)	2302 (69.5)	Ref	Ref
		Sometimes	274 (6.1)	36 (13.1)	238 (86.9)	1.46 (1.28-1.67)	1.34 (1.16-1.54)
		Rarely/never	356 (7.9)	20 (5.6)	336 (94.4)	1.59 (1.41-1.78)	1.38 (1.22-1.56)
	**Avoiding crowded places**						
		All the time/most of the time	3513 (78.1)	1434 (40.8)	2079 (59.2)	Ref	Ref
		Sometimes	457 (10.2)	129 (28.2)	328 (71.8)	1.21 (1.08-1.36)	1.16 (1.03-1.31)
		Rarely/never	528 (11.7)	59 (11.2)	469 (88.8)	1.50 (1.36-1.66)	1.35 (1.21-1.50)
	**Staying home when sick**						
		All the time/most of the time	3852 (85.6)	1505 (39.1)	2347 (60.9)	Ref	Ref
		Sometimes	232 (5.2)	47 (20.3)	185 (79.7)	1.31 (1.13-1.52)	1.22 (1.05-1.42)
		Rarely/Never	414 (9.2)	70 (16.9)	344 (83.1)	1.36 (1.22-1.53)	1.27 (1.13-1.42)
**Attitudes toward COVID-19**							
	**Ever tested positive for COVID-19**						
		No	4385 (97.5)	1583 (36.1)	2802 (63.9)	Ref	Ref
		Yes	113 (2.5)	39 (34.5)	74 (65.5)	1.02 (0.81-1.29)	1.05 (0.83-1.32)
	**Know someone who had COVID-19**						
		No	3162 (70.3)	1101 (34.8)	2061 (65.2)	Ref	Ref
		Yes	1336 (29.7)	521 (39)	815 (61)	0.94 (0.86-1.01)	0.98 (0.90-1.06)
	**Anticipated effect of COVID-19 on own health**						
		Mild or no symptoms	1085 (24.1)	201 (18.5)	884 (81.5)	Ref	Ref
		Manageable symptoms	1940 (43.1)	747 (38.5)	1193 (61.5)	0.75 (0.69-0.82)	0.80 (0.73-0.86)
		Severe symptoms	1026 (22.8)	452 (44.1)	574 (55.9)	0.69 (0.62-0.76)	0.73 (0.66-0.82)
		Possible death	447 (9.9)	222 (49.7)	225 (50.3)	0.62 (0.53-0.72)	0.65 (0.56-0.76)
	**Concern for friends/family getting sick**						
		Not concerned	1204 (26.8)	165 (13.7)	1039 (86.3)	Ref	Ref
		Concerned	3294 (73.2)	1457 (44.2)	1837 (55.8)	0.65 (0.60-0.70)	0.70 (0.64-0.76)
	**Live with someone who is high risk for COVID-19**						
		No	2649 (58.9)	874 (33)	1775 (67)	Ref	Ref
		Yes	1849 (41.1)	748 (40.5)	1101 (59.5)	0.89 (0.82-0.96)	0.89 (0.83-0.97)

^a^Prevalence ratio is the prevalence of vaccine hesitancy compared with the prevalence of planning to take a COVID-19 vaccine.

^b^Adjusted for sex, age, province of residence, household income, education level, ethnicity, and political leaning.

^c^N/A: not applicable.

^d^Ref: reference value.

### Attitudes Toward COVID-19

A small proportion of participants had tested positive for COVID-19 (113/4498, 3%) and almost one-third (1336/4498, 29.7%) knew someone who had tested positive for COVID-19 (Table 3). Participants who were concerned about their friends or family getting sick from COVID-19 had lower prevalence of vaccine hesitancy compared with those who were not concerned (aPR 0.70, 95% CI 0.64-0.76). Participants who reported living with an individual at high risk had lower prevalence (aPR 0.89, 95% CI 0.83-0.97) of vaccine hesitancy. Compared with participants who anticipated experiencing mild or no symptoms in the event of contracting COVID-19, participants had lower prevalence of vaccine hesitancy if they reported they anticipated experiencing manageable symptoms (aPR 0.80, 95% CI 0.73-0.86), severe symptoms (aPR 0.73, 95% CI 0.66-0.82), or possible death (aPR 0.65, 95% CI 0.56-0.76).

### Trusted Sources of COVID-19 Information and Institutions

Participants who trusted chief medical officers of health (aPR 0.54, 95% CI 0.47-0.61) and public health websites (aPR 0.68, 95% CI 0.59-0.77) had lower prevalence of vaccine hesitancy compared with participants who did not (Table 4). Those who reported trusting internet searches for COVID-19 information had a higher prevalence of vaccine hesitancy compared to those who did not (aPR 1.34, 95% CI 1.21-1.49). Participants who reported that their most trusted social media platform was Reddit (aPR 0.64, 95% CI 0.51-0.80) had lower vaccine hesitancy than those who did not trust this source. We found that distrust in health care institutions, government, technology companies, finance industries, and professional services was associated with vaccine hesitancy (Table 4). Participants who reported that they did not trust government institutions had higher prevalence of vaccine hesitancy (aPR 1.61, 95% CI 1.46-1.78) compared with those who reported trust in government institutions. The prevalence of hesitancy was also higher in those who did not trust health care (aPR 1.43, 95% CI 1.25-1.62), technology (aPR 1.22, 95% CI 1.08-1.38), and finance (aPR 1.14, 95% CI 1.03-1.25) compared with those who reported trust in these institutions.

**Table 4:** Associations between COVID-19 vaccine hesitancy, trusted sources of COVID-19 information, and trust in institutions in October to November 2020 (N=4498)^a^.

Sources			Total (N)	Vaccine hesitancy, n (%)			Prevalence ratio^b^ (95% CI)		Adjusted prevalence ratio^c^ (95% CI)
				No	Yes				
**Most trusted sources for COVID-19 information**									
	Chief Medical Officer of Health		1933	904 (46.8)	1029 (53.2)	0.76 (0.70-0.82)		0.80 (0.73-0.86)	
	Public health websites		1754	778 (44.4)	976 (55.6)	0.83 (0.77-0.90)		0.86 (0.80-0.94)	
	Health care provider		1239	514 (41.5)	725 (58.5)	0.91 (0.84-1.00)		0.93 (0.85-1.01)	
	Television/radio news		607	233 (38.4)	374 (61.6)	0.98 (0.88-1.10)		0.96 (0.86-1.07)	
	Internet searches (eg, Google)		529	81 (15.3)	448 (84.7)	1.43 (1.29-1.58)		1.34 (1.21-1.49)	
	Friends and family		159	33 (20.8)	126 (79.2)	1.28 (1.07-1.53)		1.16 (0.97-1.39)	
	Print newspaper		134	55 (41)	79 (59)	0.94 (0.75-1.18)		0.96 (0.77-1.21)	
**Most trusted social media platforms for COVID-19 information**									
	Facebook		2167	778 (35.9)	1389 (64.1)	1.00 (0.93-1.08)		0.98 (0.91-1.06)	
	YouTube		976	297 (30.4)	679 (69.6)	1.12 (1.02-1.22)		1.08 (0.99-1.17)	
	Twitter		797	342 (42.9)	455 (57.1)	0.87 (0.79-0.96)		0.91 (0.82-1.00)	
	Instagram		450	144 (32)	396 (88)	1.07 (0.95-1.21)		1.09 (0.96-1.22)	
	Reddit		407	196 (48.2)	211 (51.8)	0.79 (0.69-0.91)		0.84 (0.72-0.97)	
**Trust in institutions**									
	**Health care**								
		Trust	3370	1440 (42.7)	1930 (57.3)	Ref^d^		Ref	
		Neutral	828	154 (18.6)	674 (81.4)	1.42 (1.30-1.55)		1.33 (1.22-1.45)	
		Do not trust	300	28 (9.3)	272 (90.7)	1.58 (1.39-1.80)		1.43 (1.25-1.62)	
	**Government**								
		Trust	1401	744 (53.1)	657 (46.9)	Ref		Ref	
		Neutral	1414	569 (40.2)	845 (59.8)	1.27 (1.15-1.41)		1.24 (1.12-1.38)	
		Do not trust	1683	309 (18.4)	1374 (81.6)	1.74 (1.59-1.91)		1.61 (1.46-1.78)	
	**Technology**								
		Trust	579	261 (45.1)	318 (54.9)	Ref		Ref	
		Neutral	1707	648 (38)	1059 (62)	1.13 (1.00-1.28)		1.15 (1.01-1.30)	
		Do not trust	2212	713 (32.2)	1499 (67.8)	1.23 (1.09-1.39)		1.22 (1.08-1.38)	
	**Finance**								
		Trust	1325	537 (40.5)	788 (59.5)	Ref		Ref	
		Neutral	1824	641 (35.1)	1183 (64.9)	1.09 (1.00-1.19)		1.09 (0.99-1.19)	
		Do not trust	1349	444 (32.9)	905 (67.1)	1.12 (1.03-1.24)		1.14 (1.03-1.25)	

^a^Participants could pick more than one most trusted source from each list.

^b^Prevalence ratio is the prevalence of vaccine hesitancy compared with the prevalence of planning to take a COVID-19 vaccine.

^c^Adjusted for sex, age, province of residence, household income, education level, ethnicity, and political leaning.

^d^Ref: reference value.

### Cluster Analysis

**Figure 1:**
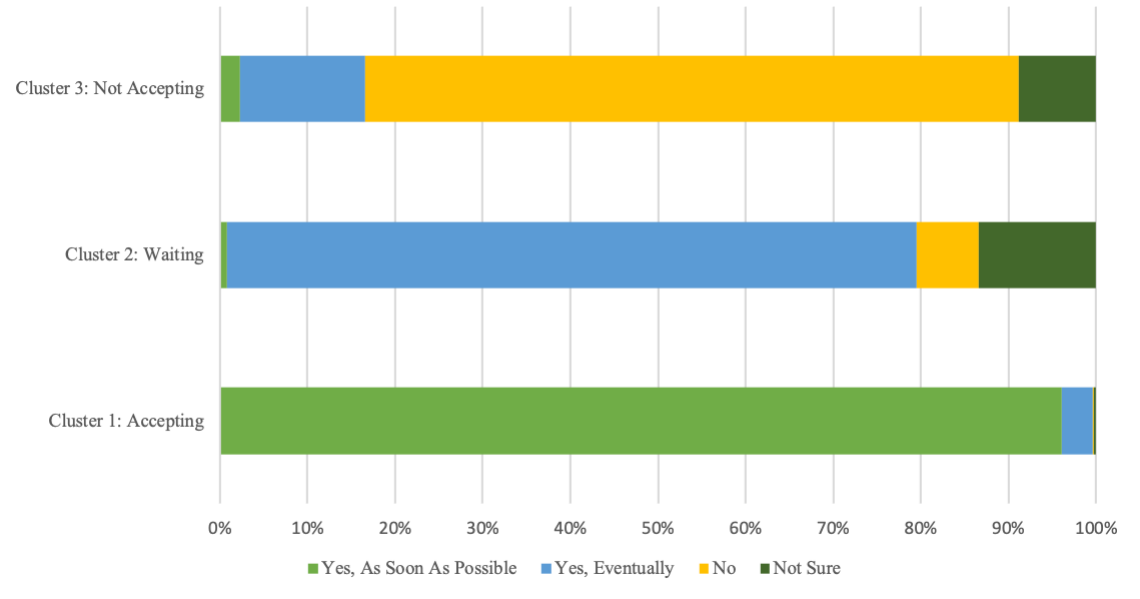
Willingness of the survey participants, by cluster, to take the COVID-19 vaccine when available.

Three distinct nonoverlapping clusters were identified through cluster analysis (Multimedia Appendix 2). Cluster 1 (the vaccine and NPI-accepting cluster) consisted of 1652 (36.7%) participants who reported willingness to take a COVID-19 vaccine and adherence to NPIs, including physical distancing, wearing a face mask in public spaces, staying home when sick, and avoiding public spaces. The 2099 (46.7%) participants in Cluster 2 (the vaccine waiting and NPI accepting cluster) also reported adherence to NPIs but were vaccine hesitant, with 1652 (78.7%) reporting that they would eventually get a vaccine; however, they planned to wait a while (Figure 1). Cluster 3 (the vaccine and NPI nonaccepting cluster) consisted of 747 (16.6%) participants who reported less adherence to NPIs and were vaccine hesitant, with 557 (74.6%) reporting that they would not take a COVID-19 vaccine when offered (Table 5).

**Table 5:** Participant characteristics by cluster (N=4498) in October to November 2020.

Characteristic			Total, n (%)		Cluster, n (%)							Prevalence ratio^a^ (95% CI)		
					Cluster 1: Accepting		Cluster 2: Waiting		Cluster 3: Not accepting		Cluster 1 versus Cluster 2			Cluster 3 versus Cluster 2
Participants			4498 (100)		1652 (36.7)		2099 (46.7)		747 (16.6)		N/A^b^			N/A
**Sex at birth**														
	Female	2294 (51)		837 (36.5)		1171 (51)		286 (12.5)		Ref^c^			Ref	
	Male	2204 (49)		815 (37)		928 (42.1)		461 (20.9)		1.12 (1.02-1.24)			1.69 (1.46-1.96)	
**Age (years)**														
	18-34	1341 (29.8)		511 (38.1)		621 (46.3)		209 (15.6)		Ref			Ref	
	35-54	1585 (35.2)		516 (32.5)		741 (46.8)		328 (20.7)		0.91 (0.80-1.03)			1.22 (1.02-1.45)	
	55+	1572 (34.9)		625 (39.8)		737 (46.9)		210 (13.3)		1.02 (0.890-1.14)			0.88 (0.73-1.06)	
**Province of residence**														
	Alberta	1998 (44.4)		684 (34.2)		836 (41.8)		478 (23.9)		Ref			Ref	
	British Columbia	502 (11.2)		185 (36.9)		273 (54.4)		44 (8.8)		0.90 (0.76-1.06)			0.38 (0.28-0.52)	
	Prairie provinces^d^	445 (9.9)		158 (35.5)		215 (48.3)		72 (16.2)		0.94 (0.79-1.12)			0.69 (0.54-0.88)	
	Ontario	800 (17.8)		318 (39.7)		404 (50.5)		78 (9.8)		0.98 (0.86-1.12)			0.44 (0.35-0.57)	
	Quebec	502 (11.2)		202 (40.2)		250 (49.8)		50 (10)		0.99 (0.85-1.16)			0.46 (0.34-0.61)	
	Atlantic provinces^d^	251 (5.6)		105 (41.8)		121 (48.2)		25 (10)		1.03 (0.84-1.27)			0.47 (0.31-0.70)	
**Household income^e^ (CAD)**														
	<$50,000	1030 (22.9)		341 (33.1)		532 (51.7)		157 (15.2)		Ref			Ref	
	$50,000-$99,999	1353 (30.1)		489 (36.1)		644 (47.6)		220 (16.3)		1.10 (0.96-1.27)			1.12 (0.91-1.37)	
	$100,000-$199,999	1300 (28.9)		532 (40.9)		559 (43)		209 (16.1)		1.25 (1.09-1.43)			1.19 (0.97-1.47)	
	≥$200,000	229 (5.1)		100 (43.7)		87 (38)		42 (18.3)		1.37 (1.10-1.71)			1.43 (1.02-2.01)	
	Rather not say	586 (13)		190 (32.4)		277 (47.3)		119 (20.3)		1.04 (0.87-1.24)			1.32 (1.04-1.67)	
**Highest level of education**														
	High school graduate or less	897 (19.9)		262 (29.2)		433 (48.3)		202 (22.5)		Ref			Ref	
	Some college or trade school	840 (18.7)		240 (28.6)		421 (50.1)		179 (21.3)		0.96 (0.81-1.15)			0.94 (0.77-1.15)	
	College or trade school	996 (22.1)		304 (30.5)		491 (49.3)		201 (20.2)		1.01 (0.86-1.20)			0.91 (0.75-1.11)	
	Some university	454 (10.1)		190 (41.8)		197 (43.4)		67 (14.8)		1.30 (1.08-1.57)			0.80 (0.61-1.05)	
	University degree	1311 (29.2)		656 (50)		557 (42.5)		98 (7.5)		1.43 (1.24-1.66)			0.47 (0.37-0.60)	
**Ethnicity**														
	Caucasian	3862 (85.9)		1455 (37.7)		1765 (45.7)		642 (16.6)		Ref			Ref	
	Indigenous/First Nations/Metis/Inuit	228 (5.1)		71 (31.1)		107 (46.9)		50 (21.9)		0.88 (0.70-1.12)			1.19 (0.90-1.59)	
	Asian	193 (4.3)		67 (34.7)		115 (59.6)		11 (5.7)		0.81 (0.64-1.04)			0.33 (0.18-0.59)	
	Caribbean/African/South American	70 (1.5)		16 (22.9)		43 (61.4)		11 (15.7)		0.60 (0.37-0.98)			0.76 (0.42-1.39)	
	Other	145 (3.2)		43 (29.6)		69 (47.6)		33 (22.8)		0.85 (0.63-1.15)			1.21 (0.85-1.72)	
**Political leaning**														
	Liberal	1841 (40.9)		971 (52.7)		821 (44.6)		49 (2.7)		1.48 (1.30-1.68)			0.29 (0.21-0.40)	
	Moderate/middle of the road	1029 (22.9)		327 (31.8)		565 (54.9)		137 (13.3)		Ref			Ref	
	Conservative	1628 (36.2)		354 (21.7)		713 (43.8)		561 (34.5)		0.91 (0.78-1.05)			2.26 (1.87-2.72)	

^a^Determined using negative binomial regression.

^b^N/A: not applicable.

^c^Ref: reference value.

^d^Prairie provinces include Saskatchewan and Manitoba; Atlantic provinces include Nova Scotia, New Brunswick, Prince Edward Island, and Newfoundland and Labrador.

^e^CAD $1=US $0.75.

Compared with participants in Cluster 2 (the vaccine waiting and NPI-accepting cluster), participants in Cluster 3 (the vaccine and NPI nonaccepting cluster) were more likely to be male (PR 1.69, 95% CI 1.46-1.96), 35-54 years of age (PR 1.22, 95% CI 1.02-1.45), have a household income of CAD $200,000 (USD $150,200; CAD $1=US $0.75) or more (PR 1.32, 95% CI 1.04-1.67), and report a conservative political leaning (PR 2.26, 95% CI 1.87-2.72). Participants in Cluster 1 (the vaccine and NPI-accepting cluster) were more likely to be Liberal leaning (PR 1.48, 95% CI 1.30-1.68), have some university education (PR 1.30, 95% CI 1.08-1.57), or a university degree (PR 1.43, 95% CI 1.24-1.66), have an annual household income of CAD $100,000-$199,999 (PR 1.25, 95% CI 1.09-1.43) or CAD $200,000 or more (PR 1.37, 95% CI 1.10-1.71), and male (PR 1.12, 95% CI 1.02-1.24) compared with participants in Cluster 2 (the vaccine-waiting and NPI-accepting cluster).

## Discussion

### Principal Findings

In this national cross-sectional survey completed in the fall of 2020 prior to the approval of COVID-19 vaccines in Canada, we found that 63.9% (2876/4498) of the participants reported COVID-19 vaccine hesitancy, ranging from delaying vaccine administration when offered to not planning to take a vaccine. Vaccine hesitancy was associated with several sociodemographic factors including (1) younger age (18-39 years), (2) lower education, and (3) non-Liberal political leaning. Participants who reported vaccine hesitancy had higher prevalence of reporting being concerned about vaccine side effects, did not believe that a COVID-19 vaccine would end the pandemic or that the benefits of a COVID-19 vaccine outweighed the risks, and had lower prevalence of reporting being influenced by peers or health care professionals. We identified 3 distinct participant clusters: (1) participants who reported adherence to NPIs and did not have vaccine hesitancy, (2) individuals who reported adherence to NPIs but did have vaccine hesitancy, and (3) individuals who reported less adherence to NPIs and vaccine hesitancy.

The 3 distinct clusters of vaccine acceptance can inform targeted vaccination campaign messaging in a novel way by directing messages to address cluster-specific concerns with respect to vaccine hesitancy. The majority of the participants in Cluster 2 (the vaccine waiting and NPI-accepting cluster) planned to delay taking a vaccine when offered, while the majority in Cluster 3 (the vaccine and NPI nonaccepting cluster) did not intend to take a vaccine at all. Messaging related to preventing COVID among friends and family, highlighting the benefits, and ensuring health care workers are knowledgeable and supported in their vaccine counselling may be more helpful for those in Cluster 2 relative to those in Cluster 3. Participants in Cluster 3 were more likely to be male, 35-54 years of age, have an annual household income of CAD $200,000 or more, report Conservative political leaning, and live in Alberta compared with participants in Cluster 2. The characteristics of Cluster 3 are consistent with current trends in vaccine uptake in that less uptake has been seen among Albertans, males, and individuals aged 18 to 59 years as of October 23, 2021 [17]. Based on our findings, Cluster 3 appears quite challenging to target messaging toward and further qualitative research is needed to determine how best to target this subgroup of vaccine-hesitant individuals to increase vaccine uptake.

As of October 27, 2021, more than 1,700,000 Canadians have been infected with COVID-19 and more than 28,000 Canadians have died [1]. Reported intention to get vaccinated has been variable [18-23], and as supply of vaccine outweighs demand among eligible individuals within Canada [24], there are growing concerns about vaccine hesitancy with respect to COVID-19 vaccines. The Government of Canada reports that as of October 23, 2021, 29,613,930 (77%) individuals 12 years of age or older had received 1 dose of COVID-19 vaccine and 28,086,337 (73%) were fully vaccinated [17]. Vaccine hesitancy among Canadians has decreased since the time our survey was administered, which is likely multifactorial. A recent qualitative study in the United States found that vaccine uptake among individuals who were initially vaccine hesitant is related to 3 factors: (1) intrinsic factors (eg, protecting oneself from COVID-19), (2) extrinsic factors (eg, protecting family or friends), and (3) structural factors (eg, vaccine mandates) [25].

While there has been a decrease in vaccine hesitancy over time, many of the underlying predictors of hesitancy have remained stable over time [5,7,10,26-29]. Many studies [10,19-21,30,31] have reported that female sex at birth was associated with COVID-19 vaccine hesitancy [18]. We found that lower education level was associated with COVID-19 vaccine hesitancy. Both low [10,19,20,30] and high [32] education level have been associated with COVID-19 vaccine hesitancy, while lower household income has more consistently been associated with vaccine hesitancy [10,18,30]. The conflicting associations between these sociodemographic factors and vaccine hesitancy suggest that these associations may be region-specific on a global scale as was identified by Lazarus et al [32] or may be time-dependent, as these cross-sectional surveys were completed at different points of time in the COVID-19 pandemic.

We did not find an association between ethnicity and COVID-19 vaccine hesitancy, although an association has been reported in several other studies [19,29,30,33]. In a qualitative study, Momplaisir et al [33] found several themes that contributed to vaccine hesitancy among individuals who identified as Black, including mistrust in the medical community, racial injustice, and COVID-19–specific concerns, including the speed of development and concerns about potential side effects. This highlights that COVID-19 vaccine hesitancy is complex with many contributing factors, all of which need to be addressed to effectively combat vaccine hesitancy and encourage individuals to take a COVID-19 vaccine when offered. Although population segments that are more likely to be vaccine hesitant can be identified and messages can be tailored to those population segments, the content and delivery of the messaging needs to consider the complex interaction of all the domains of the SAGE working group vaccine hesitancy determinant framework (ie, contextual influences, vaccine characteristics, and individual/social group influences) [8]. Messaging needs to be designed in collaboration with these population segments through partnership-based community-embedded work to address the complex and unique circumstances contributing to vaccine hesitancy.

The influences of COVID-19 vaccine characteristics and administration of COVID-19 vaccines on vaccine hesitancy are unique compared to annual influenza campaigns or childhood immunization schedules. In response to the COVID-19 pandemic, the scientific community has come together to develop safe and effective vaccines [2]. At the time of survey administration, prior to the regulatory approval of COVID-19 vaccines in Canada, we found that almost half of the respondents were concerned that these vaccines had been developed too quickly and the majority were concerned about the short- and long-term vaccine side effects. These concerns about COVID-19 vaccines have been reported in other studies [19,31,33], and we found that they were associated with vaccine hesitancy. We also found that COVID-19 vaccine hesitancy was associated with lower concern about the consequences of becoming infected with COVID-19 or concern about family or friends becoming infected. Vaccination campaigns need to address these COVID-19–specific factors in their messaging.

Trust in government has been identified as a factor associated with acceptance of a COVID-19 vaccine [18]. Consistent with this, we found that vaccine hesitancy was associated with a lack of trust in government and health care institutions. When developing messaging to combat COVID-19 vaccine hesitancy, it is important to consider the importance of trust, which has been highlighted in previous pandemics, including the H1N1 pandemic [34]. To improve trust and consistency of messaging, supportive programs need to be available for health care workers to build knowledge and confidence in their messaging. The trusted sources of COVID-19 information should also be considered when designing targeted vaccination campaigns.

### Limitations

The major limitation of this cross-sectional study was that it represents one snapshot in time in the fall of 2020 prior to the approval of COVID-19 vaccines in Canada and as the country was entering the second wave of the pandemic; therefore, the responses provided by participants at that time have evolved. The survey recruited participants from an existing voluntary nationwide panel designed to be representative of the Canadian population; however, by using a panel, there will be a component of selection bias as participants have volunteered to partake in research surveys through an electronic platform, which may lead to increased selection of individuals with higher socioeconomic status or education level leading to an underestimation of vaccine hesitancy. We included all provinces and territories in our sampling strata; however, we did oversample Alberta, which could lead to bias in the results and make these findings less generalizable to the Canadian population. To minimize this bias, province of residence was included in all adjusted analyses. Response bias should also be considered as individuals who chose to respond to the web-based survey may differ systematically from those who chose not to respond.

### Conclusion

COVID-19 vaccines are an important tool in the fight against the COVID-19 pandemic; yet, vaccine hesitancy is a concern. We have identified population segments that are associated with vaccine hesitancy (eg, younger age, lower education level) that can be targeted with public health messaging as well as attitudes toward COVID-19, COVID-19 vaccines, and NPIs that can inform messaging content. Effectively addressing vaccine hesitancy is important to increase vaccine uptake.

## Appendix

Multimedia Appendix 1 Survey questions.

Multimedia Appendix 2 Cluster analysis.

## Abbreviations

aPR: adjusted prevalence ratio
NPI: nonpharmacologic intervention
PR: prevalence ratio
SAGE: Strategic Advisory Group of Experts
STROBE: Strengthening Reporting of Observational Studies in Epidemiology
