# Supplementary material for: COVID-19 Vaccine–Related Attitudes and Beliefs in Canada: National Cross-sectional Survey and Cluster Analysis
Source: JMIR Public Health Surveill. 2021 Dec 23;7(12):e30424. doi: 10.2196/30424 (PMC8709417; doi:10.2196/30424)
Supplement: Multimedia Appendix 1 [file publichealth_v7i12e30424_app1.docx]

Behavior change / vaccine uptake study

We have some questions about the COVID-19 pandemic. We want to better understand Canadians’ beliefs, and practices and knowledge regarding public health actions aimed at preventing the spread of COVID-19. As always, all your responses are confidential.

**Q1.**

**Base=All**

**Multichoice**

Thinking about the last couple of weeks of your own life, which of the following words best describe what you have been feeling the most? Please pick up to three.

[Randomize list]

Optimistic

Happy

Healthy

Grateful

Energized

Supported

Lonely

Anxious

Bored

Angry

Unmotivated

Unhealthy

**Q2.**

**Base=All**

**Rating Scale 1 – 5**

Thinking about the following statement, how much would you say each describes you, personally, on a scale from 1 to 5?

[Randomize Rows]

Religion is very important in my day-to-day life

I think it is important to have new experiences that challenge you

I prioritize time with family above all else

I prefer to express my views even if others don’t agree

I value saving money whenever I can

I consider my impact on the environment in most things I do

I appreciate celebrating diversity

Tradition is an important part of my life

It is important for me to keep up with the latest trends in lifestyle and fashion

It’s important to me that I find ways to express myself creatively

**Q3.**

**Base = All**

**Single Choice**

Have you or anyone you know tested positive for COVID-19?

Yes, I have

Yes, someone I know

No, neither

**Q4.**

**Base=All**

**Single Choice**

How much do you believe your own health would suffer if you got COVID-19?

I would be able to cope easily with the disease - relatively mild, just minor symptoms

I think I would have serious but manageable symptoms – like a case of the flu

I think I would be very sick with severe symptoms – I have some risks

I think I could possibly die from the disease – I am high risk

**Q5.**

**Base=All**

**Single Choice**

How concerned are you about your friends or family becoming sick from COVID-19

Very concerned

Concerned

Not that concerned

Not at all concerned

**Q6.**

**Base=All**

**Single Choice**

Some people might be at “high risk” for COVID-19 if they are older, if their immune system is compromised, or if they have diabetes, heart disease, or obesity. Do you live with anyone that is “high risk” for COVID-19?

Yes

No

**Q7.**

**Base=All**

**Single choice**

In the past few weeks, how many people outside your household have you regularly interacted with? Your best guess is fine.

1-5 people

6-10 people

11-15 people

16-20 people

More than 20 people

**Q8.**

**Base=All**

**Single choice grid**

And over the past few weeks, **how often** have you been doing each of the following:

Social distancing (also called physical distancing). That is, keeping at least 2 metres distance from other people who are not in your social bubble

Wore a face covering in public indoor spaces when you couldn’t keep physical distance from others

Avoiding places & activities where you would interact with a large number of people outside your household

Staying home if you were sick with any symptoms, even mild ones

All the time

Most of the time

Sometimes

Rarely

Never

**Q9.**

**Base=All**

**Single choice grid**

And **how effective** do you believe each of the following are at reducing the spread of the virus in your community?

Social distancing (also called physical distancing)

Wearing a face covering in public indoor spaces

Avoiding places & activities where you would interact with a large number of people outside your household

Staying home if you were sick with any symptoms, even mild ones

Very effective

Pretty effective

Not that effective

Very ineffective

Not sure/Can’t say

**Q10.**

**Base=All**

**Single choice grid**

Has the messaging or advertising you’ve seen, read or heard messaging from local, provincial or federal health officials had an impact on your likelihood to do any of these?

Social distancing (also called physical distancing)

Wearing a face covering in public indoor spaces

Avoiding places & activities where you would interact with a large number of people outside your household

Staying home if you were sick with any symptoms, even mild ones

Made me much more likely to do this

Made me a little more likely to do this

Has made no difference one way or the other

Has made me a little less likely to do this

Had made me a lot less likely to do this

I have not seen any messaging or advertising from the local, provincial, or federal health officials

**Q11.**

**Base=Rarely or never to any in Q8**

**Multichoice**

Over the past few weeks, when you have not followed public health recommendations, what were the reasons (check all that apply)

I intended to, but I simply forgot

While I feel following recommendations are important, following them was too burdensome

I do not think following recommendations is important for my health

I do not think following recommendations is important for the health of my friends/family

I don’t believe recommendations work

The people around me don’t follow recommendations

Other (Please specify)

OK, onto a related issue: COVID-19 vaccines. Right now, there is no COVID-19 vaccine available, but doctors and public health officials believe one or more may be available next year.

**Q12.**

**Base=All**

**Single choice**

If a vaccine against COVID-19 became available to you, what would you do:

Get a vaccine as soon as possible

Eventually get a vaccine, but wait a while first

NOT get a vaccine

Not sure

**Q13.**

**Base=NO and Not sure in Q12**

**Open text**

If you would not get a vaccine as soon as possible, why not?

**Q14.**

**Base=all**

**Single choice grid**

For each statement, please indicate whether you agree or disagree:

[rows] [randomize]

I would get a vaccine to protect my family

A COVID-19 vaccine will end the pandemic

In a normal year I usually try to get the flu vaccine

I’m concerned about short-term side effects of a COVID-19 vaccine

I’m concerned about longer-term side effects of a COVID-19 vaccine

I don’t trust the COVID-19 vaccine because of how fast it is being developed

The benefits of a COVID-19 vaccine would outweigh its risks

I’m more likely to get a COVID-19 vaccine if my friends and family do

If my family doctor / pharmacist / public health nurse tell me to get the vaccine I am much more likely to do so

Strongly agree

Agree

Disagree

Strongly disagree

Not sure/Can’t say

**Text Screen**

**[AB respondents ONLY]**

OK, we now have some questions around apps to prevent the spread of COVID-19

ABTraceTogether is an app that works in Alberta

Here’s how it works: When you’re within a two-meter range of someone else for more than 15 minutes, both phones exchange random codes through Bluetooth pings. If someone else with the app is diagnosed with COVID-19, a contact tracer can see who that person was in contact with and notify you that you may have been exposed. The app does not use GPS and does not collect information about where you have been.


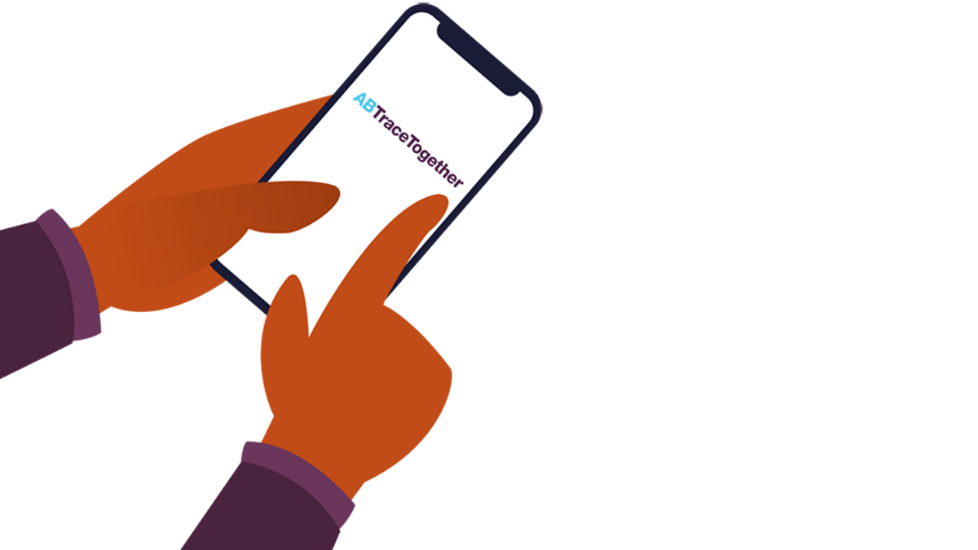


**[Rest of Canada, exclude AB]**

OK, we now have some questions around apps to prevent the spread of COVID-19

COVID Alert is an app that works in most provinces and territories

Here’s how it works: When you’re within a two-meter range of someone else for more than 15 minutes, both phones exchange random codes through Bluetooth. The codes cannot be used to identify you or them. If someone else with the app is later diagnosed with COVID-19, they can choose to upload this information to the app confidentially. If they do you’ll then be notified through your phone that you may have been exposed. The app does not use GPS and does not collect information about where you have been.


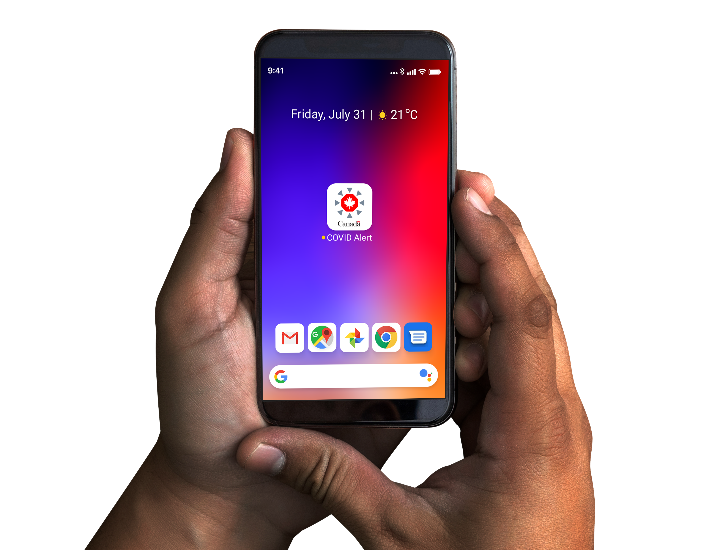

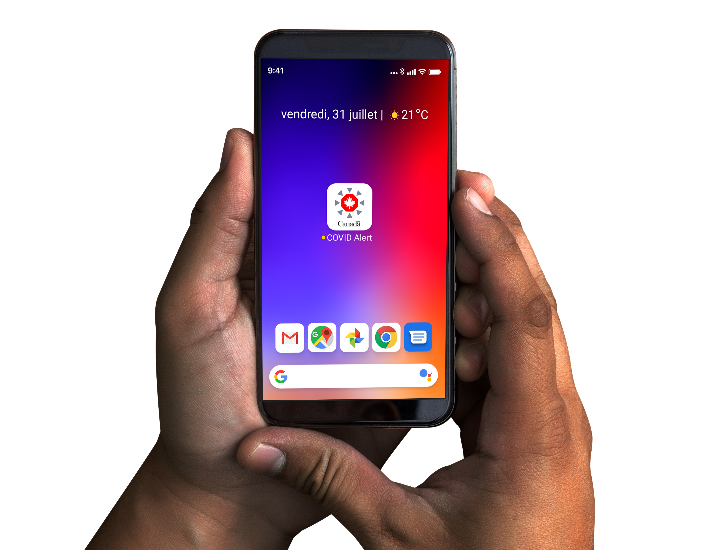


**Q15.**

**Base=All**

**Single choice**

Have you downloaded ABTraceTogether[AB] / COVID Alert[ROC] at any point?

Yes

No

**Q16.**

**Base=No in Q15**

**Multichoice**

Why have you not downloaded the app? Please select all that apply:

Did not know about it

Concerned about privacy

Do not trust the government not to collect my data

Worried about battery usage on my phone

The app collects too much data

Don’t think it’s going to be effective

Not enough cases in my area

Not in contact with others enough to need it

Don’t know how to download it

Don’t own a smartphone OR smartphone is too old for the app

**Q17**

**Base=AB ONLY**

**Single choice**

The Alberta government is currently considering the opportunities and implications of transitioning to the federal COVID Alert app, including optimizing its effectiveness for Albertans and ensuring that the privacy and security of Albertans’ health information is safeguarded.

Will you download COVID Alert when it starts working in Alberta?

Yes

No

Maybe

Not Sure

**Q18**

**Base=Rest of Canada/No in Q15**

**Single choice**

If you do not have COVID Alert, will you download it in the future?

Yes

No

Maybe

Not sure

**Q19**

**Base=All**

**Single choice**

For a contact tracing app to work effectively, people who test positive need to voluntarily enter a code provided by public health to let the app know to notify their contacts.

If you have the app and test positive, would you enter your unique code into the app?

Yes

No

Maybe

Don’t know

**Q20.**

**Base=all**

**Single choice grid**

For each statement, please indicate whether you agree or disagree:

[rows] [randomize]

Public health messaging around COVID-19 has been clear and easy to understand

Public health messaging around COVID-19 has been consistent

COVID-19 restrictions are significantly harming our economy

Younger people are mostly to blame for the increase in COVID-19 cases

There’s too much focus on how COVID-19 affects older people

**Q21.**

**Base=All**

**Multichoice**

What, if any, social media platforms do you use? (Check all that apply).

TikTok

Reddit

YouTube

Instagram

Facebook

Twitter

Snapchat

Twitch

Dating apps (e.g., Tinder, Bumble, Hinge)

Other

None

**Q22.**

**Base=Exclude None in Q21**

**Multichoice**

Of the platforms you use, which do you trust the most for COVID-19 information?

(select up to three)

Pipe in list of those selected in Q21

**Q23.**

**Base=All
Multichoice**

Which of the following do you use for COVID-19 information? Select all that apply

Physician or other healthcare provider (pharmacist/nurse etc.)

Public health websites, either local, provincial, or federal

Chief Medical Officer of Health Media Briefings (Federal or Provincial)

Friends and family

Television/radio news

Print newspaper

Google and other internet searches

None

**Q24.**

**Base=Exclude None in Q23**

**Multichoice**

Of the sources you use, which do you trust the most for COVID-19 information?

(select up to three)

Pipe in list of those selected in Q23

**Q25.**

**Base=All**

**Single choice grid**

To what extent do you trust or not trust each of the following types of institutions?

[ROWS - RANDOMIZE]

Technology (i.e. Apple, Facebook, Google, etc.)

Financial (i.e. banks, insurance, investment companies, etc.)

Retail (i.e. food/beverage, clothing, personal care, etc.)

Professional services (i.e. legal, accounting, etc.)

Healthcare (i.e. hospitals, clinics, etc.)

Government

5 - Completely trust

4

3

2

1 – Do not trust at all

**Demographics**

**What is your age?**

Input number

**Are you…?**

Male

Female

Prefer to self-describe

**What is your highest level of education achieved?**

Elementary/grade school

Some high school

High school graduate

Some college/technical school

Some university

University undergraduate degree

Some post-graduate school

Post-graduate degree

**What is your household income?**

Less than 25k

25k to less than 50k

50k to less than 75k

75k to less than 100k

100k to less than 1125k

125k+

DK/Prefer not to say

**What is your postal code?**

Input postal code

**What is your ethnicity?**

British Isles (e.g. English, Irish, Scottish)

French

Other European (e.g. German, Russian, Italian, Norwegian)

Aboriginal (e.g. Inuit, Metis, North American Indian)

Other North American (e.g. Canadian, American, Newfoundlander, Quebecoise)

Caribbean (e.g. Jamaican, Barbadian, Cuban, West Indian)

Latin, Central and South American (e.g. Mexico, Argentinian, Guatemalan, Peruvian)

African (e.g. South African, Ethiopian, Nigerian, Zimbabwean)

Arab/West Asian (e.g. Lebanese, Moroccan, Iranian, Turk)

South Asian (e.g. East Indian, Pakistani, Goan, Sri Lankan)

Chinese

Other East and South East Asian (e.g. Fillipino, Vietnamese, Korean, Japanese)

Jewish (non-denominational)

Other

**Do you identify as a visible minority?**

Yes

No

**What is your marital status?**

Married

Living common law

Separated / Divorced

Widowed

Never married

**What is your current employment status?**

Employed / self-employed full-time (30 or more hours per week)

Employed / self-employed part-time (fewer than 30 hours per week)

Student

Homemaker

Retired

Currently looking for work

Not working for medical reasons

Other

**Do you have children under the age of 18 years living in your house?**

Yes

No

**What is your living situation? Check all that apply**

Alone

With children

With parents

With other family members

With non-family roommates

Other

**How would you describe your political leaning?**

Very liberal

Liberal

Slightly liberal

Moderate/middle-of-the-road

Slightly conservative

Conservative

Very conservative
