# Supplementary material for: COVID-19 Vaccine–Related Attitudes and Beliefs in Canada: National Cross-sectional Survey and Cluster Analysis
Source: JMIR Public Health Surveill. 2021 Dec 23;7(12):e30424. doi: 10.2196/30424 (PMC8709417; doi:10.2196/30424)
Supplement: Multimedia Appendix 2 [file publichealth_v7i12e30424_app2.docx]

|  |  | **Clusters** |  |  |
| --- | --- | --- | --- | --- |
|  | **1 (N=2099)** | **2 (N=1652)** | **3 (N=747)** | **Overall (N=4498)** |
| **Social distancing** |  |  |  |  |
| Mean (SD) | 1.7 (± 0.69) | 1.6 (± 0.65) | 2.9 (± 1.1) | 1.8 (± 0.89) |
| **Face cover** |  |  |  |  |
| Mean (SD) | 1.3 (± 0.63) | 1.2 (± 0.49) | 3.1 (± 1.4) | 1.5 (± 1.0) |
| **Avoiding public places** |  |  |  |  |
| Mean (SD) | 1.6 (± 0.81) | 1.5 (± 0.79) | 3.5 (± 1.3) | 1.9 (± 1.2) |
| **Staying home** |  |  |  |  |
| Mean (SD) | 1.4 (± 0.96) | 1.3 (± 0.84) | 2.6 (± 1.5) | 1.6 (± 1.1) |
| **factor(vaccine)** |  |  |  |  |
| 1 Yes | 17 (1 %) | 1588 (96 %) | 17 (2 %) | 1622 (36 %) |
| 2 Yes but wait | 1652 (79 %) | 58 (4 %) | 107 (14 %) | 1817 (40 %) |
| 3 No | 150 (7 %) | 1 (0 %) | 557 (75 %) | 708 (16 %) |
| 4 Maybe | 280 (13 %) | 5 (0 %) | 66 (9 %) | 351 (8 %) |
| **Agreement on statements about vaccine** | | |  |  |
| **Protect my family** | 2.0 (± 0.69) | 1.2 (± 0.43) | 3.3 (± 0.77) | 1.9 (± 0.95) |
| **End the pandemic** | 2.7 (± 0.69) | 2.4 (± 0.71) | 3.4 (± 0.72) | 2.7 (± 0.79) |
| **Normally get flu shot** | 2.4 (± 1.1) | 1.6 (± 0.90) | 3.3 (± 0.98) | 2.3 (± 1.2) |
| **Short-term side effects** | 2.0 (± 0.71) | 2.8 (± 0.79) | 1.8 (± 0.92) | 2.3 (± 0.87) |
| **Long-term side effects** | 1.9 (± 0.71) | 2.7 (± 0.82) | 1.6 (± 0.90) | 2.2 (± 0.91) |
| **Don’t trust due to**  **speed of development** | 2.1 (± 0.73) | 3.2 (± 0.67) | 1.6 (± 0.84) | 2.4 (± 0.94) |
| **Benefits outweigh risks** | 2.3 (± 0.62) | 1.5 (± 0.63) | 3.4 (± 0.79) | 2.2 (± 0.92) |
| **Influence of family/friends** | 2.5 (± 0.75) | 2.4 (± 0.93) | 3.5 (± 0.67) | 2.7 (± 0.90) |
| **Influence of health professional** | 2.2 (± 0.81) | 1.5 (± 0.71) | 3.4 (± 0.74) | 2.2 (± 1.0) |
|  |  |  |  |  |
|  |  |  |  |  |
|  |  |  |  |  |
|  |  |  |  |  |
|  |  |  |  |  |
|  |  |  |  |  |
|  |  |  |  |  |
|  |  |  |  |  |
|  |  |  |  |  |
|  |  |  |  |  |

|  |  | **Clusters** |  |  |
| --- | --- | --- | --- | --- |
| Mean (SD) | **1 (N=2099)** | **2 (N=1652)** | **3 (N=747)** | **Overall (N=4498)** |
| **Effective and clarify of public messaging Q9 and Q10** | | | | |
| Social distancing effectiveness | 1.6 (± 0.62) | 1.4 (± 0.57) | 2.7 (± 0.84) | 1.7 (± 0.78) |
| Face cover effectiveness | 1.7 (± 0.75) | 1.4 (± 0.58) | 3.5 (± 0.71) | 1.9 (± 0.99) |
| Avoiding public spaces effectiveness | 1.3 (± 0.54) | 1.2 (± 0.47) | 2.6 (± 0.88) | 1.5 (± 0.78) |
| Staying home when sick effectiveness | 1.2 (± 0.49) | 1.1 (± 0.38) | 1.9 (± 0.91) | 1.3 (± 0.61) |
| Social distancing messaging | 1.8 (± 0.97) | 1.6 (± 0.89) | 2.8 (± 0.78) | 1.9 (± 1.0) |
| Face cover messaging | 1.7 (± 0.91) | 1.5 (± 0.83) | 2.8 (± 0.78) | 1.8 (± 0.98) |
| Avoiding public spaces messaging | 1.8 (± 0.94) | 1.6 (± 0.91) | 2.9 (± 0.73) | 1.9 (± 1.0) |
| Staying home when sick messaging | 1.7 (± 0.95) | 1.6 (± 0.89) | 2.6 (± 0.95) | 1.8 (± 0.99) |
|  | | | | |
| **Reason for not following public health recommendations (Q11)** | | | | |
| Forgot | 0.041 (± 0.20) | 0.029 (± 0.17) | 0.078 (± 0.27) | 0.043 (± 0.20) |
| Too burdensome | 0.022 (± 0.15) | 0.013 (± 0.11) | 0.098 (± 0.30) | 0.031 (± 0.17) |
| Not important for my health | 0.0038 (± 0.062) | 0.0042 (± 0.065) | 0.15 (± 0.36) | 0.029 (± 0.17) |
| Not important for health of friends/family | 0.00048 (± 0.022) | 0.00061 (± 0.025) | 0.11 (± 0.31) | 0.019 (± 0.14) |
| Recommendations do not work | 0.0014 (± 0.038) | 0.0036 (± 0.060) | 0.44 (± 0.50) | 0.074 (± 0.26) |
| Others around me do not follow them | 0.010 (± 0.10) | 0.010 (± 0.10) | 0.078 (± 0.27) | 0.022 (± 0.15) |
| Other reason | 0.033 (± 0.18) | 0.025 (± 0.16) | 0.18 (± 0.38) | 0.054 (± 0.23) |

|  |  | **Cluster** |  |  |
| --- | --- | --- | --- | --- |
| (Q3-7) | **1 (N=2099)** | **2 (N=1652)** | **3 (N=747)** | **Overall (N=4498)** |
| Concern for own health | 2.3 (± 0.88) | 2.4 (± 0.88) | 1.5 (± 0.70) | 2.2 (± 0.91) |
| Concern for family/friends | 1.9 (± 0.77) | 1.7 (± 0.68) | 3.2 (± 0.77) | 2.0 (± 0.91) |
| Tested positive for COVID | 0.026 (± 0.16) | 0.025 (± 0.16) | 0.023 (± 0.15) | 0.025 (± 0.16) |
| Know someone who tested positive for COVID | 0.30 (± 0.46) | 0.33 (± 0.47) | 0.22 (± 0.41) | 0.30 (± 0.46) |
| Know no one who tested positive for COVID | 0.68 (± 0.47) | 0.65 (± 0.48) | 0.76 (± 0.42) | 0.68 (± 0.47) |
| Live with high-risk individual | 1.6 (± 0.49) | 1.5 (± 0.50) | 1.7 (± 0.45) | 1.6 (± 0.49) |
| Interactions outside household | 2.1 (± 1.4) | 2.1 (± 1.3) | 3.3 (± 1.6) | 2.3 (± 1.5) |
